# Effect of Spent Coffee Grounds on the Crystallinity and Viscoelastic Behavior of Polylactic Acid Composites

**DOI:** 10.3390/polym15122719

**Published:** 2023-06-17

**Authors:** Anne Shayene Campos de Bomfim, Daniel Magalhães de Oliveira, Kelly Cristina Coelho de Carvalho Benini, Maria Odila Hilário Cioffi, Herman Jacobus Cornelis Voorwald, Denis Rodrigue

**Affiliations:** 1 Fatigue and Aeronautical Materials Research Group, Department of Materials and Technology, UNESP-São Paulo State University, Guaratinguetá 12516-410, SP, Brazil; daniel.m.oliveira@unesp.br (D.M.d.O.); kcccbenini@gmail.com (K.C.C.d.C.B.); odila.cioffi@unesp.br (M.O.H.C.); h.voorwald@unesp.br (H.J.C.V.); 2 Center for Research on Advanced Materials (CERMA), Department of Chemical Engineering, Université Laval, Quebec, QC G1V 0A6, Canada; denis.rodrigue@gch.ulaval.ca

**Keywords:** polylactic acid (PLA), spent coffee grounds (SCG), biocomposites, rheological properties, mechanical properties

## Abstract

This work investigated the addition of spent coffee grounds (SCG) as a valuable resource to produce biocomposites based on polylactic acid (PLA). PLA has a positive biodegradation effect but generates poor proprieties, depending on its molecular structure. The PLA and SCG (0, 10, 20 and 30 wt.%) were mixed via twin-screw extrusion and molded by compression to determine the effect of composition on several properties, including mechanical (impact strength), physical (density and porosity), thermal (crystallinity and transition temperature) and rheological (melt and solid state). The PLA crystallinity was found to increase after processing and filler addition (34–70% in the 1st heating) due to a heterogeneous nucleation effect, leading to composites with lower glass transition temperature (1–3 °C) and higher stiffness (~15%). Moreover, the composites had lower density (1.29, 1.24 and 1.16 g/cm^3^) and toughness (30.2, 26.8 and 19.2 J/m) as the filler content increased, which is associated with the presence of rigid particles and residual extractives from SCG. In the melt state, polymeric chain mobility was enhanced, and composites with a higher filler content became less viscous. Overall, the composite with 20 wt.% SCG provided the most balanced properties being similar to or better than neat PLA but at a lower cost. This composite could be applied not only to replace conventional PLA products, such as packaging and 3D printing, but also to other applications requiring lower density and higher stiffness.

## 1. Introduction

Polylactic acid (PLA) is a well-known biodegradable thermoplastic polymer and is vastly used for packaging and 3D printing applications. It is polymerized by polycondensation or ring-opening polymerization from the lactic acid monomer. Moreover, lactic acid can be fermented from natural sugars such as glucose or sucrose, including the sugars from spent coffee grounds fermentation [1]. The PLA molecular structure and molar mass directly affect the polymer’s properties. In particular, thermal properties, such as melting temperature and glass transition temperature, are strongly related to the molecular structure, as well as the thermo-mechanical history of the polymer [2]. Despite its positive effect on the environment due to its biodegradability, PLA is reported to have low ductility, toughness, resistance to moisture, rate of crystallization, and glass transition temperature [3].

To improve the properties, several efforts have been made to fill PLA with different natural fillers as a disperse phase to modify the polymer chain mobility and consequently enhance its properties [3,4,5,6]. Most of the PLA have low crystallinity (3–10%), and filler addition was reported to significantly improve PLA crystallinity by reorganizing the polymeric chains and influencing the crystallization rate via heterogeneous nucleation. For example, PLA filled with sisal (10 wt.%) was compared with polypropylene (PP) composites [7]. The results showed that the PLA composite had a 270% increase in crystallinity, while the PP composite was only improved by 12%. Moreover, a recent review concluded that PLA with improved properties could be developed by using different natural fillers, depending on the application requirements, since natural fibers are diversified and abundant [8].

Spent coffee grounds (SCG), as a residue of coffee beverages, is a valuable resource rich in polysaccharides, polyphenols, and proteins being widely studied for different applications, including composite filler [9]. A recent review discussed the valorization of SCG for biopolymers synthesis and as composites filler, highlighting the great versatility of SCG in improving different polymers properties [10]. Other works investigated PLA filled with different SCG concentrations and treatments to compare with other fillers and additives, showing promising results in improving PLA properties and biodegradability [11,12,13,14]. Although various techniques have been used to characterize the composites, none of the works focused on the effect of SCG addition on the material’s crystallinity and viscoelastic properties. Combined dynamic mechanical analysis (DMA) and rheology, performed respectively in the solid and melt state of PLA filled with SCG, were not found in the current literature.

Moreover, PLA/SCG composites are a recent topic of research. According to the Scopus database, only 21 documents were published on this subject between 2013 and 2023 (May). The data show an increased tendency over the years. This subject is achieving scientific relevance, considering the number of citations peak in 2022 (177) [15].

Thus, the main objective of this work is to investigate the effect of spent coffee grounds content (0, 10, 20, and 30% wt.) on PLA crystallinity, as well as to determine the effect of crystallinity on the thermo-mechanical and viscoelastic properties of the composites produced via extrusion followed by compression molding. Although some previous works discussed the subject of PLA/SCG composites, the novelty of this work focuses on the characterizations being done in both the solid and melt state after SCG addition, as crystallinity and viscoelastic proprieties, which directly affect PLA processing and applications, are important for their applications.

## 2. Materials and Methods

### 2.1. Materials

Pellets of PLA 2003D were supplied from NatureWorks (Minnetonka, MN, USA), while the spent coffee grounds (SCG) were collected as a post-consumer residue (mixture) from coffee shops on the campus of Laval University. Raw SCG was previously dried in an oven at 60 °C for a week to ensure that the moisture was completely removed.

### 2.2. Composite Preparation

**Figure 1:**
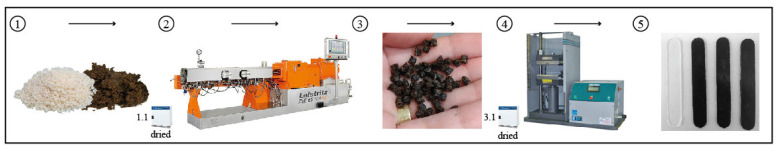
Preparation of the composites: (1) PLA pellets and raw SCG, (2) the extruder, (3) the composites’ extruded pellets, (4) the hydraulic press, and (5) the obtained specimens.

The materials were first dried in an oven at 60 °C for 24 h and then mixed in a twin-screw extruder Leistritz ZSE-27 (Remscheid, Germany) having a screw diameter of 27 mm diameter, a L/D ratio (length/diameter) of 40 and 10 heating zone. The PLA pellets were fed in the first zone of the extruder, while the SCG was fed in the fourth zone via a side stuffer. The composition was determined by controlling the specific flow rate for each component (PLA and SCG) by calculating the weight/time ratio (SCG = 0, 10, 20, and 30 wt.%). The extruder was operated at the same constant mass flow rate of 100 g/min and a screw speed of 60 rpm for each composition. The temperature profile in the extruder was controlled at 175 °C (zone 1), 185 °C (zones 2–9), and 165 °C (last zone). A circular die (2.7 mm in diameter) was used to form the melt into a filament shape. The materials were cooled in a water bath close to the die exit (about 10 cm) and then pelletized using a model 304 pelletizer (Conair, Stanford, CT, USA). Then, the pellets were dried in an oven at 60 °C for 48 h before use. Finally, the pellets were molded in an SC7620 automatic hydraulic press (Carver, Wabash, IN, USA) at 210 °C for 7 min with a constant force of 2 tons and compressing pressure in the mold of 0.5 MPa. The mold and plates were cooled with water circulation for about 3 min until getting to 70 °C. The plates were produced (210 × 210 × 2.4 mm^3^) and cut into different specimens for characterization, as described next. The preparation of the composites is illustrated in Figure 1.

### 2.3. Characterization

#### 2.3.1. Differential Scanning Calorimetry (DSC)

Dual scan analysis was carried out on a DSC Q20 (TA Instruments, New Castle, DE, USA) with a sample weight of around 5 mg in a temperature range from 0 to 200 °C under constant nitrogen flow (40 mL/min). The heating and cooling rate was 10 °C/min. Melting and cold crystallization temperatures (*T_m_* and *T_cc_*), as well as melting and cold crystallization enthalpies (Δ*H_m_* and Δ*H_cc_*) and crystallization degree (*X_c_*) were determined from the thermograms (heat flow as a function of temperature). The crystallinity degree was calculated as:

(1) $$X_{c=}{}_{\left[\frac{\Delta H_{m}-\Delta H_{cc}}{f\;\times \;\Delta H_{m100\%\;}}\right]\;100}$$

where Δ*H_m_*_100%_ is the enthalpy of a fully crystalline PLA sample (93.7 J/g), while f is the weight fraction of PLA (matrix) in the samples [16,17,18].

#### 2.3.2. Thermo-Gravimetric Analysis (TGA)

TGA was carried out on SII Nanotechnology INC equipment model Exstar 6000—TG/DTA series (Tokyo, Japan) with a sample weight of around 10 mg. The analysis was performed in a platinum pan under a constant nitrogen flow (100 mL/min) in a temperature range from 30 to 600 °C and a heating rate of 10 °C/min. Thermal degradation temperature (*T_onset_*) was determined via DTG curves where an inflection point was observed in the baseline.

#### 2.3.3. Dynamic Mechanical Analysis (DMA)

DMA was carried out on a RSA3 (TA Instruments, New Castle, DE, USA) using rectangular specimens (82.3 × 12.5 × 2.4 mm^3^) cut from the molded plates. A three-point bending geometry was selected with a span of 40 mm. Two types of characterization were performed after strain sweep verification to stay in the linear viscoelastic range of the materials. First, temperature sweeps were done from 25 to 80 °C with a heating rate of 0.5 °C/min and a frequency of 1 Hz. Then, frequency sweeps were performed at room temperature (23 °C) between 0.01 and 25 Hz with a strain of 0.01%.

#### 2.3.4. Rheology

The shear rheological properties in the melt state were studied using a rotational rheometer (ARES, Rheometric Scientific, TA Instruments, New Castle, DE, USA) with a parallel plate geometry (25 mm in diameter). All the analyses were performed under a nitrogen atmosphere. First, strain sweep tests (0.01 to 100%) were performed at a frequency of 1 Hz to determine the linear viscoelastic zone of the samples. Then, frequency sweeps (0.01 to 40 Hz) were performed at a deformation of 5% using three temperatures: 180, 190, and 200 °C.

#### 2.3.5. Impact Test

The Charpy impact strength was performed on a manual impact testing machine Wolfgang Ofist using a 4 J pendulum, type A. Seven rectangular “V” notched specimens (82.3 × 12.5 × 2.4 mm^3^) of PLA and its composites were tested at room temperature according to ASTM D6110. The “V” notch was produced by a manual milling machine Vigorelli (type 1), using a bi-angular milling cutter.

#### 2.3.6. Density and Porosity

The density of PLA and the composites (*ρ_r_*) was measured by a helium gas pycnometer Ultrapyc 1200e (Quantachrome Instruments, Boynton Beach, FL, USA) at room temperature. The samples were previously dried in an oven at 60 °C for 24 h and weighed. To calculate the porosity, the apparent density ($\rho_{a}$) of the samples was measured via the Archimedes principle using water and calculated as:

(2) $$\rho_{a}=\frac{w_{0\;}\rho_{f}}{w_{0}-w_{1}}$$

where $\rho_{f}$ is the density of the fluid (water), while *w*_0_ and *w*_1_ are the samples’ weight in air and immersed in water, respectively.

The porosity of the samples was calculated from the real (*ρ_r_*) and apparent density (*ρ_a_*) as:

(3) $$Porosity=\left(1-\frac{\rho_{a}}{\rho_{r}}\right)\times 100$$

#### 2.3.7. Scanning Electron Microscopy

Morphological analysis of the composites was carried out on the fractured surface of samples after impact testing. The specimens were previously dried and fixed on metal support using carbon adhesive tape before being coated (metalized) with gold. The images were taken with a Zeiss EVO LS-15 scanning electron microscopy (Cambridge, UK) combined with EDS/EBDS Oxford INCA Energy 250 system (Oxford Instruments, Oxfordshire, UK) operating at 5 kV.

#### 2.3.8. Statistical Analysis

One-way ANOVA and grouping information using the Tukey method to calculate the statistical significance of impact test results were carried out using the statistical software Minitab 18.1 (Minitab Inc., State College, PA, USA). A significance index of 95% (*p*-value < 0.05) was used.

## 3. Results and Discussions

### 3.1. DSC and TGA

**Figure 2:**
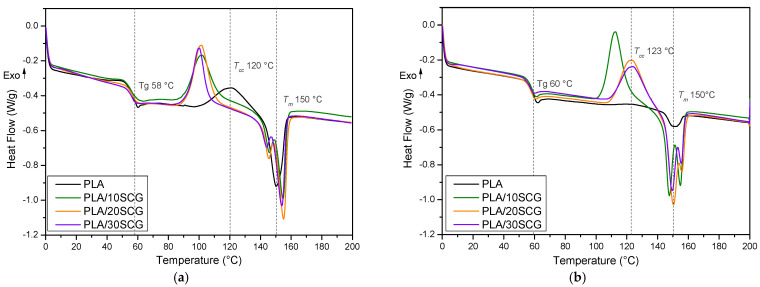
DSC curves for PLA and the composites: (**a**) first heating and (**b**) second heating.

The DSC curves are presented in Figure 2, and the thermal parameters are summarized in Table 1. The first heating scan eliminates the thermal history from PLA and its composites, providing different values from the second heating scan. The thermal history is directly related to the compression molding conditions, mainly the cooling rate [19]. The cooling scan did not present thermal events, but a crystallization peak appeared in the heating scan, called cold crystallization. According to Table 1, PLA melting temperature (*T_m_*) was determined as the endothermic peak at around 150 °C, while cold crystallization temperature (*T_cc_*) occurred at around 122 °C. However, *T_cc_* was not observed in the second heating scan (Figure 2b) due to a slow crystallization rate and low crystallinity degree (3.4%). For the composites, SCG addition generated the presence of two *T_m_* in both heating scans. These peaks are related to the melting of the original crystals and the newly formed crystals due to the melt-recrystallization process or the presence of a dual lamellae structure formed by the various heating scans [20]. Moreover, T_cc_ was well-defined around 112–123 °C for the composites in both heating scans. This shows that the polymeric chains were more organized after SCG addition due to a heterogeneous nucleation effect [21]. This observation can be confirmed by the significant improvement (34–70% in the 1st heating) of the composites’ crystallinity compared to the neat PLA, especially in the second heating scan (about 1500%) (Table 1). The literature also reported PLA crystallinity increases after SCG addition, but the differences were not as high (93% at 15 wt.% and 66% at 20 wt.%) [11,22]. Although composites usually present higher crystallinity as the filler content increases, there is always a maximum related to chain mobility and spatial hindering. In our case, the maximum (70.3%) was observed for PLA/20SCG. This trend can also be related to particle agglomeration in PLA/30SCG acting as defects.

Finally, the glass transition temperature (T_g_) of neat PLA was found to be around 59 °C in the first and second heating scans. This result highlights that the thermal processing of the specimens did not significantly modify the PLA structure (degradation), leading to similar T_g_ of the matrix and the composites in the first and second heating scans. However, the composites presented slightly lower T_g_ values (2–3 °C difference) than the neat PLA in both scans due to higher polymer chain mobility associated with the presence of rigid particles releasing extractives from SCG. A similar trend of decreasing T_g_ was reported for PLA composites using treated SCG [22] and PLA with waste paper [23]. Another work used PLA with grapevine biochar (1 and 10 wt.%) and concluded that the filler acted as a nucleating agent by increasing the crystallinity and reducing the T_g_ values of the composites [24]. However, different behavior was identified for PLA/SCG (1–7 wt.%) 3D printed composites in which the Tg was the same for all samples, mostly influenced by the filler content and the processing conditions [25]. These results show the effect of SCG content, as well as the extrusion/compression steps, on the PLA’s crystallinity and chain mobility. Although the presence of rigid fillers should limit polymer chain mobility, SCG extractives can be released, leading to increased mobility as well as acting as nucleating agents/plasticizers, especially for a higher SCG concentration.

**Figure 3:**
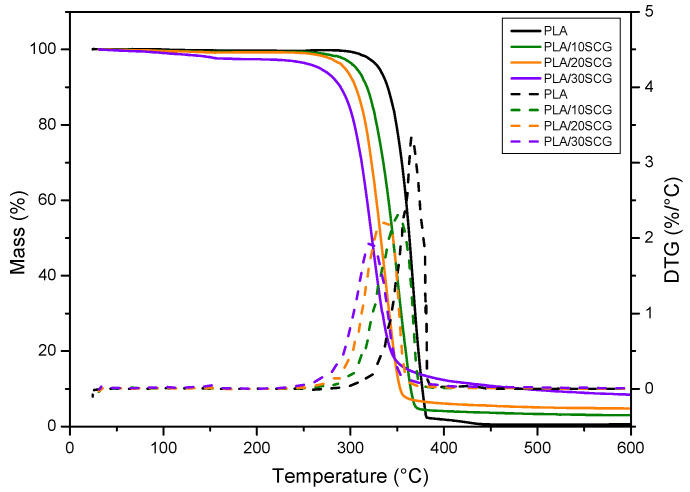
TGA curves for PLA and the composites.

TGA curves are presented in Figure 3, and the thermal parameters are presented in Table 1. The thermal degradation temperature (T_onset_) was found to decrease as the filler content decreased (11% for PLA/10SCG and 21% for PLA/30SCG). This behavior is not in agreement with the results reported by previous works, such as PLA filled with SCG [26,27], PLA filled with rice straw hydrochar [28], and polypropylene (PP) filled with SCG [29,30]. Moreover, the T_onset_ values show that the composites were not degraded in the extrusion/compression processing (T < 210 °C). Residual weight at 600 °C also increased with increasing filler content, which is related to extractives, mostly polysaccharides [31], and ashes from the natural filler. Some works reported that SCG residual weight was in the range of 5–25% [31,32].

### 3.2. Dynamic Mechanical Analysis (DMA)

**Figure 4:**
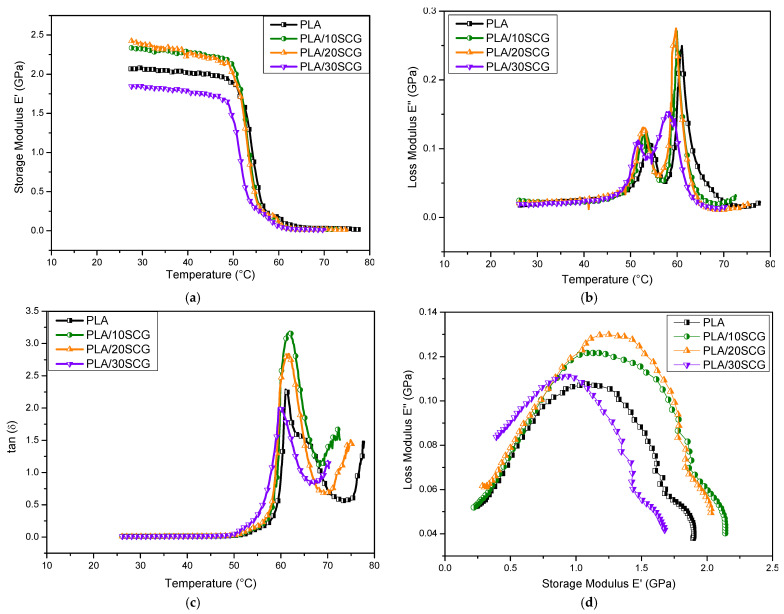
DMA curves for PLA and its composites: (**a**) storage modulus as a function of temperature, (**b**) loss modulus as a function of temperature, (**c**) tan (δ) as a function of temperature, and (**d**) Cole–Cole plots.

In the solid state, DMA results are presented in terms of storage modulus (E′), loss modulus (E″), and tan (δ) (= E″/E′) in Figure 4. Figure 4a shows that for E′, the values at 30 °C were selected for comparison. In this case, PLA/10SCG and PLA/20SCG presented higher values (2.32 and 2.39 GPa, respectively) than PLA/30SCG (1.84 GPa) and PLA (2.07 GPa), indicating that composites with intermediate SCG contents (10–20%) are slightly stiffer and store more energy than neat PLA and 30 wt.% SCG. This trend can be related to the DSC results (Table 1), showing that PLA/20SCG presented the highest crystallinity. Similar storage modulus increases were reported for injection-molded PLA composites filled with SCG and bamboo (30 wt.%) [32] and PLA composites filled with nanocellulose from sugarcane bagasse (1–5 wt.%) [33]. However, recent work reported that PLA/SCG (20 wt.%) with and without lactic acid oligomers had a lower storage modulus compared to neat PLA, which was associated with a plasticization effect caused by the SCG remaining oil [26]. Around 50–60 °C, the storage modulus significantly decreases for all samples, which corresponds to the glass transition range where the material significantly losses stiffness. For the loss modulus (E″), Figure 4b shows that the maximum energy dissipation can be determined from the maximum on the E″ curve. By increasing the SCG content, the peak temperature slightly decreased from 61.0 °C to 58.2 °C. Moreover, two transitions were identified in E″ curves. The first one (51–55 °C) corresponds to a secondary transition related to the motion of localized links (bending and stretching) and the relaxation of lateral groups from the polymeric chain, while the second transition (58–61 °C) corresponds to the glass transition temperature (T_g_). Finally, the damping factor (tan δ) followed the moduli trends: chain mobility restriction caused by SCG addition directly influenced the damping properties of the polymer [34]. Gonzalez-Lopez et al. (2019) observed a similar trend in PLA composites filled with agave fibers (10–30 wt.%) and stated that lower tan (δ) with increasing filler concentration may be related to better polymer/filler interaction/adhesion [35]. Other studies supported this observation, such as PLA/polyester/kenaf laminated composite and PLA/silk composite [36,37]. As reported by Hassan et al. (2014), the tan δ peak shifts to higher temperatures, combined with a decreased peak intensity, indicating a restriction of molecular mobility due to improved interactions between the filler and matrix [38].

Cole–Cole plots (E″ as a function of E′) are presented in Figure 4d. It was noticed that semicircular curves are associated with homogenous systems and well-dispersed fillers in a polymeric matrix [33,39]. According to the curves, PLA/10SCG shows the most semicircular arc, similar to PLA, indicating a homogenous composite and good SCG dispersion. However, higher filler content has a more irregular curve, especially for PLA/30SCG. This indicates a more heterogenous structure with poorly dispersed filler. This is also related to lower crystallinity (Table 1) and lower E′ (Figure 4b) compared to the other composites.

**Figure 5:**
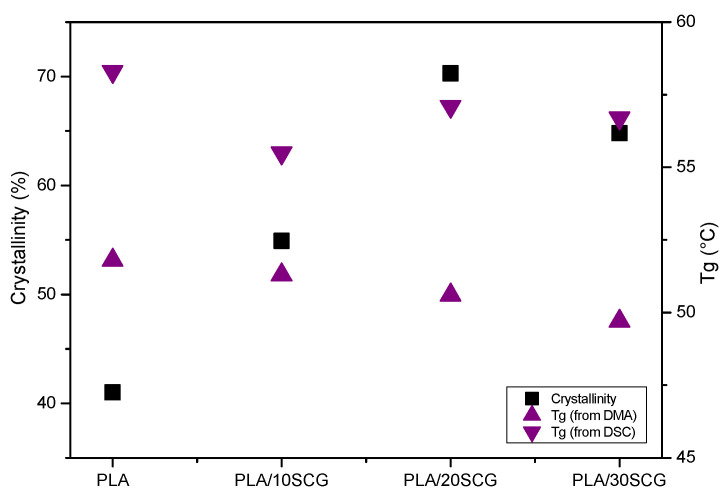
PLA crystallinity (DSC) and T_g_ (DSC and DMA) as a function of SCG content.

To complete the analysis, Figure 5 compares the glass transition temperature (T_g_) from DSC (1st heating) and DMA. The T_g_ from DMA was determined from E′ curves whose value decreased as the filler content increased: 51.8 °C for PLA, 51.3 °C for PLA/10SCG, 50.6 °C for PLA/20 SCG, and 49.7 °C for PLA/30SCG. This trend is also observed in the T_g_ obtained by DSC, emphasizing the effect of filler addition, especially with increasing filler content [40]. The relation between T_g_ and crystallinity as a function of filler content is also presented in Figure 5d. It was clearly identified that as the crystallinity increased, the T_g_ from DSC and DMA decreased, supporting the SCG effect observed on the polymeric chains.

### 3.3. Rheology

**Figure 6:**
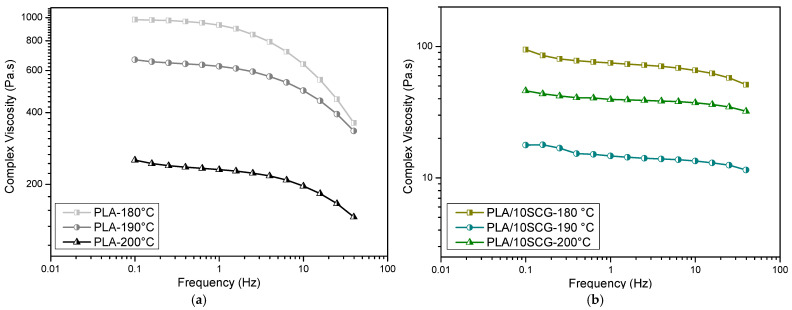

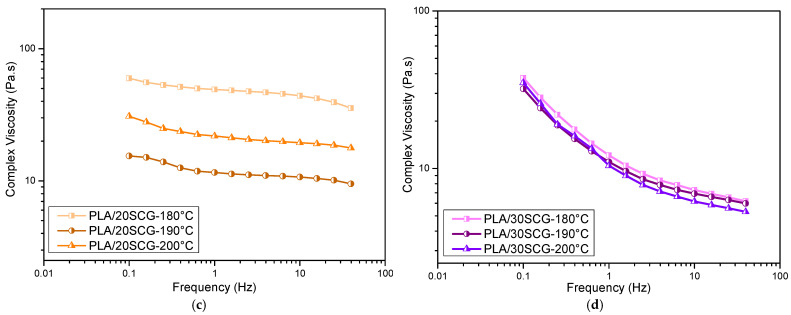
Complex viscosity as a function of frequency at different temperatures (180, 190, and 200 °C) for (**a**) PLA, (**b**) PLA/10SCG, (**c**) PLA/20SCG, and (**d**) PLA/30SCG.

**Figure 7:**
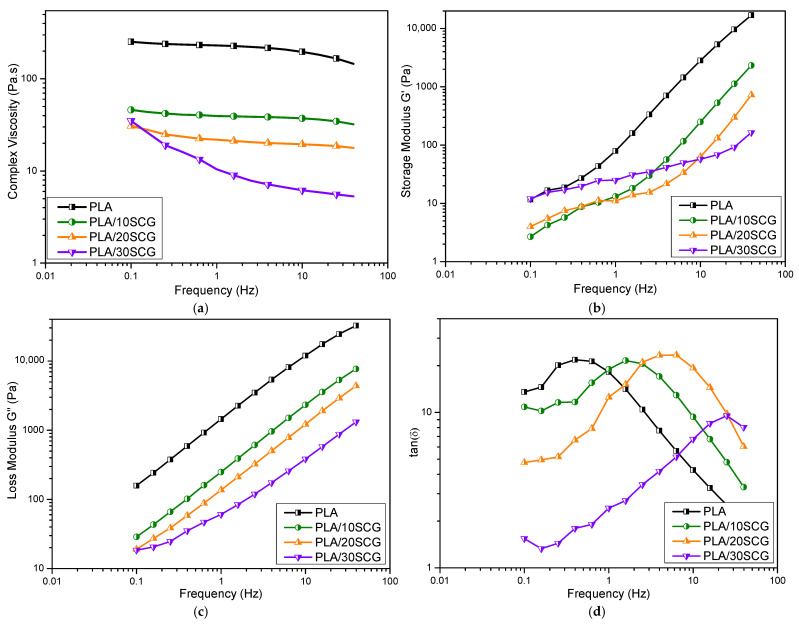
Viscoelastic properties as a function of frequency (200 °C) for PLA and the composites: (**a**) complex viscosity, (**b**) storage modulus, (**c**) loss modulus, and (**d**) tan (δ).

The rheological properties in the melt state are reported in Figure 6 and Figure 7 in terms of complex viscosity (*), storage modulus (G′), loss modulus (G″), and damping factor (tan δ = G″/G′). Figure 6 is used to determine the effect of temperature, while Figure 7 compares the effect of SCG content.

Figure 6 presents the complex viscosity as a function of frequency for different temperatures. As expected, the viscosity decreases with increasing temperature due to higher chain mobility and less interaction between them (more internal energy). However, the effect of frequency is different with increasing SCG content. For the neat PLA (Figure 6a), typical shear-thinning behavior for the neat polymer is observed with a Newtonian plateau at a low frequency and a power-law (decreasing trend) at a higher frequency. Then, PLA/10SCG and PLA/20SCG mainly have a Newtonian behavior (almost constant viscosity) and a slight shear-thinning behavior at a higher frequency (above 10 Hz). This behavior can be related to several factors, including complex interactions. First, the presence of rigid SCG particles modifies the chain mobility, especially as filler content increases.

The second factor is that higher filler content generates more shear in the extruder, possibly modifying the PLA molecular structure (more polymer degradation). However, this effect should be limited since negligible variations of the thermal properties were observed via DSC (Figure 2) and DMA (Figure 4). There is also a possibility that residual molecules (extractives such as mono/polysaccharides, carbohydrates, lipids, oils, proteins, etc.) are present from the SCG particles themselves (they were only dried and not washed before use) [41], i.e., low molecular weight materials that can act as plasticizers. Actually, this can be associated with the small bump around 155 °C that can be seen in TGA curves (Figure 4). As the SCG content increases, their concentration also increases, leading to different effects/trends on the PLA behavior. The effect is more important in the melt state (Figure 6) than in the solid state (Figure 4), and this is why both characterizations are needed to obtain complete information on these samples. Finally, PLA/30SCG presents a highly non-Newtonian behavior which is related to its higher filler content. The disappearance of the Newtonian behavior (low frequency) has been associated with the presence of rigid particles having interactions between them, i.e., particle–particle contact [42].

According to Figure 7a, all the composites have a lower viscosity than the neat PLA at 200 °C. In fact, viscosity decreased as the filler content increased, which could be related to increased polymeric chain mobility in the melt state [43]. A similar trend was reported for PLA filaments filled with waste paper (5–15 wt.%) [23], PLA filled with biochar (1–7.5 wt.%) [42], and PLA filled with different waxes, such as beeswax, candelilla, carnauba, and cocoa (3–15 wt.%) [44]. A lower viscosity also suggests that the filler acted as a plasticizer from the presence of extractives, as described above [27]. This reduced viscosity would improve the processability of these materials [22].

The storage modulus (G′), loss modulus (G″) and loss tangent (tan δ) are presented in Figure 7. All the samples show that G′ and G″ increase with frequency due to their viscoelastic nature, but the neat PLA again has higher values. At high frequencies (above 10 Hz), G′ and G″ decrease with increasing filler content. In the melt state, PLA is more rigid than the composites. However, as the filler content increases, the composites become less rigid [45]. Nizamuddin et al. (2021) reported on G′ and G″ behavior for PLA/rice straw hydrochar composites (5–20 wt.%), in which the values increased with filler content justified by the reduction of chain mobility leading to higher flow resistance [28]. Another work investigated PLA filled with cocoa (3–15 wt.%) and observed that at lower frequencies, G′ increased with increasing filler content, but at higher frequencies, a reversed trend was observed as G′ decreased with filler content, which was justified by the plasticizing effect of the natural fillers extractives [44].

The value of tan (δ) can be compared to unity to determine the viscous/elastic behavior of the materials [46]. According to Figure 7d, all the samples showed increased tan (δ) at a lower frequency, followed by a decrease at a higher frequency. PLA/10SCG and PLA/20SCG behave similarly to PLA at high frequency and then decreasing tan (δ), while PLA/30SCG shows an increasing tan (δ) trend for most of the analysis performed. At lower frequency (0.1 Hz), PLA has the highest tan (δ), indicating a more viscous behavior, while at higher frequency (40 Hz), PLA, PLA/10SCG, and PLA/20SCG present a stiffer behavior and PLA/30SCG a more elastic behavior. This decreasing tan (δ) trend with increasing frequency can be associated with chemical interaction between the polymer and the filler [28]. In this case, PLA/30SCG shows poor polymer-filler interactions, which is consistent with the density/porosity results as described next.

### 3.4. Impact Strength and Density

**Figure 8:**
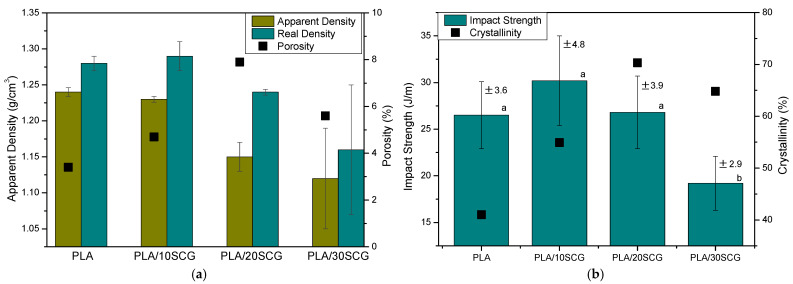
Properties of PLA as a function of SCG content: (**a**) density and porosity, and (**b**) impact strength and crystallinity.

The real and apparent densities of the composites are presented in Figure 8a. It can be seen that the composites have a lower density than PLA, especially the apparent density. The density decreased with increasing filler content as the polymer fraction was reduced, and natural fibers are known to be low-density materials. The density of SCG was reported to be 0.45 g/cm^3^ [47]. Such behavior (decreasing composites density) was also identified in PLA composites filled with nettle (10 and 25 wt.%) [48]. Furthermore, it was possible to calculate the porosity of the materials from density values. The results show that the composites have higher porosity than PLA. PLA/10SCG presented the lowest porous content (4.7%), while PLA/20SCG presented the highest value (7.9%), indicating poor dispersion and possible interfacial voids. In addition to the filler addition, the specimens’ processing also influenced the porosity of the materials since neat PLA presented 3.4% of porosity. González-López et al. (2019) also reported a decreasing density trend with increasing porosity for PLA/agave composites as the filler content increased (10–30 wt.%), but porosity values were higher than in our work: about 35% for 20 wt.% composite and 60% for 30 wt.% composite [35]. Another work used SCG with high-density polyethylene (HDPE) and found that both density and porosity increased as the filler increased (10–30 wt.%), as 55% of porosity for the 30 wt.% composite [49].

PLA has a well-known low toughness. From the PLA 2003D datasheet, a value of 16 J/m is reported [45]. Nevertheless, after extrusion/compression processing, PLA was found to have good impact strength (26.5 J/m). Although PLA/10SCG might have a slightly higher value, the composites present a slightly decreased impact strength with increasing filler content (Figure 8b) [11]. PLA filled with agave fibers (10–30 wt.%) was also reported to show lower impact strength with a reduction of 24% for 10 wt.% composite and 41% for the 30 wt.% composite [35]. In some cases, it was reported that filler addition could improve PLA toughness, but an optimum content of around 20 wt.% was observed [3]. Therefore, adding SCG could improve not only the crystallinity (DSC) and stiffness (DMA) of PLA but also its toughness (PLA/10SCG).

Statistical analysis (ANOVA) of the impact test has been carried out, and the results are shown in Table 2. As the F value (8.40) is greater than the F_critical_ value (3.01), the decision is to reject the null hypothesis (H_0_) (all means are equal) for a significance level of 0.05. In addition, considering that the *p*-value is less than the significance level (α = 0.05), the H_0_ was rejected since the *p*-value represents the probability against the null hypothesis. This result indicates that the impact strength of all the samples differs significantly. However, to determine whether the mean difference between any pair of groups is statistically significant, grouping information using the Tukey method and a significance level of 95% were carried out, which are presented with the letters a and b in Figure 8b. In these results, group a contains PLA, PLA/10SCG, and PLA/20SCG, while group b contains PLA/30SCG. The differences between the means of the PLA, PLA/10SCG, and PLA/20SCG, which share the same letter, are not statistically significant. The PLA/30SCG does not share any letters, indicating a significantly lower mean value for impact strength than the other samples.

Comparing toughness and crystallinity results, it is possible to identify that both properties increased for PLA/10SCG compared to PLA. Nevertheless, a different trend was observed for the other composites; PLA/20SCG presented the highest crystallinity leading to similar toughness compared to PLA despite presenting a higher porosity. Moreover, PLA/30SCG presented a high crystallinity but lower toughness compared to PLA, which is related to the filler content and the formation of agglomerates acting as stress concentration points, mainly for PLA/30SCG. Overall, it can be concluded that the final properties represent a balance between complex interactions.

### 3.5. Scanning Electron Microscopy

**Figure 9:**
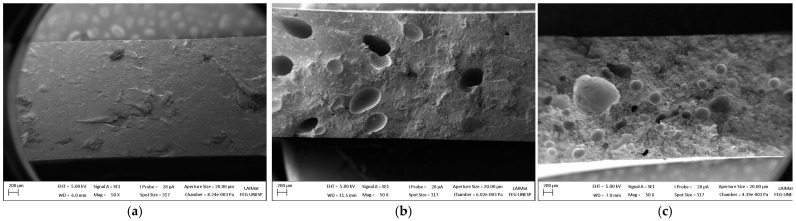
SEM images (magnification of 50×) for (**a**) PLA/10SCG, (**b**) PLA/20SCG, and (**c**) PLA/30SCG.

**Figure 10:**
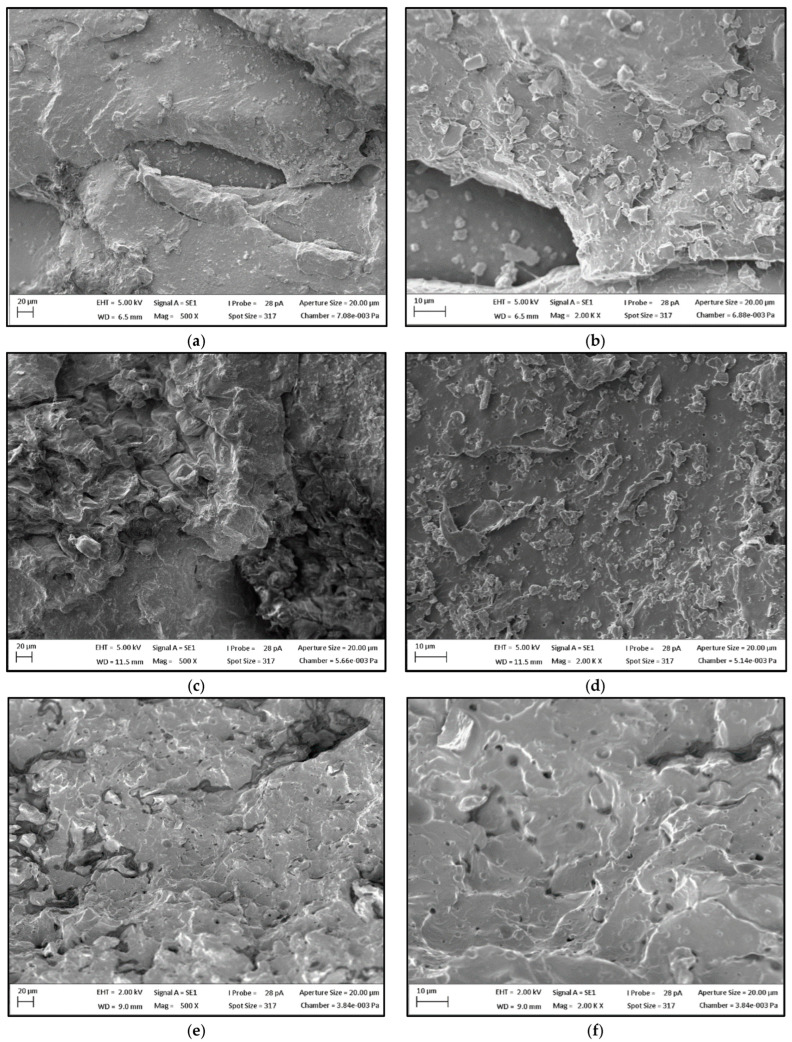
SEM images with different magnifications (500× and 2000×) for (**a**,**b**) PLA/10SCG, (**c**,**d**) PLA/20SCG, and (**e**,**f**) PLA/30SCG.

SEM images of the composites are presented in Figure 9 and Figure 10. According to these images, it is possible to identify that PLA/20SCG and PLA/30SCG presented a higher volume and size of voids than PLA/10SCG, which can be seen at lower magnification (Figure 9). These images confirm the relatively high amount of porosity reported in Figure 8 for PLA/20SCG (7.9%) and PLA/30SCG (5.6%).

In addition, small voids were observed all over the matrix for all composites, but they are more evident for PLA/20SCG and PLA/30SCG (Figure 10). It can also be seen that SCG dispersion decreases with increasing filler content as less space is available between the particles leading to more particle–particle contact and agglomeration. Although PLA/30SCG has the highest filler concentration, the particles are very challenging to identify.

It can be concluded that the composites’ morphology mostly influenced their thermo-mechanical and viscoelastic behavior, especially for PLA/20SCG and PLA/30SCG.

## 4. Conclusions

PLA and its composites based on spent coffee grounds (SCG) were successfully prepared via twin-screw extrusion and compression molding. For the range of SCG content investigated (0–30 wt.%), a series of characterizations were performed with a focus on crystallinity, thermo-mechanical (solid state), and viscoelastic (melt state) properties.

The results showed that SCG can act as nucleating agents (solid particles) and plasticizers (extractive release). This led to several differences between the neat matrix (PLA) and the composites (effect of SCG content), as well as complex interactions.

For example, the thermal processing significantly increased the PLA crystallinity, which was further improved with filler addition. Although rigid particles limit polymeric chain mobility, SCG extractives caused a higher crystallinity and lower T_g_, which is related to higher polymeric chain mobility. Furthermore, as the crystallinity increased, the density and toughness decreased as the filler content increased, while the composite with the lower filler content (10 wt.%) provided a higher toughness than PLA. However, in the melt state, polymeric chain mobility was enhanced, and composites with a higher filler content became less viscous due to the increased chain mobility. This behavior might also be associated with possible residues/extractives acting as plasticizers in the composites.

Although PLA/20SCG showed a higher porosity (7.9%), it provided the best-balanced performances having similar or even better properties than neat PLA and all the other composites. For example, higher crystallinity (38.6%), higher storage modulus at 30 °C (2.38 GPa) and at high frequencies (4.3 GPa at 10 Hz), lower viscosity (19.3 Pa·s at 10 Hz), lower density (1.16 g/cm^3^) and comparable impact strength (26.8 J/m). Nevertheless, PLA/30SCG presented high crystallinity and low density that could influence, to a greater extent, the biodegradability of the material since it was reported that the presence of a biofiller could accelerate the biodegradation process [11].

This work represents a follow-up on previous studies in our groups and the literature. These materials are interesting because they are fully biobased. In the future, more work will focus on the effect of the SCG morphology (particle geometry and size) and composition (washing, extraction, surface modification, etc.) on the final biocomposites’ properties. The effect of processing conditions (temperature, pressure, time, velocity, etc.) and method (injection molding, rotomolding, etc.) should also be compared for commercial and industrial applications. The presence of additives (coupling agents) can also be investigated to improve these results. Finally, it would be interesting to investigate the possibility of recycling and composting these materials.

## Figures and Tables

**Table 1:** Thermal parameters obtained via DSC and TGA curves.

Sample	1st Heating							2nd Heating							TGA	
	T_m1_ (°C)	T_m2_ (°C)	T_cc_ (°C)	ΔH_m_ (J/g)	ΔH_cc_ (J/g)	T_g_ (°C)	X_c_ (%)	T_m1_ (°C)	T_m2_ (°C)	T_cc_ (°C)	ΔH_m_ (J/g)	ΔH_cc_ (J/g)	T_g_ (°C)	X_c_ (%)	T_onset_ (°C)	Residue_600°C_ (%)
PLA	150.4	-	120.5	19.9	−18.5	58.3	41.0	150.0	-	-	3.2	-	59.9	3.4	291.3	0.6
PLA/10SCG	146.0	154.7	102.1	25.2	−21.1	55.5	54.9	147.7	154.9	112.5	25.6	−24.3	58.9	59.2	259.3	2.9
PLA/20SCG	145.5	155.1	101.4	28.7	−24.0	57.1	70.3	150.4	155.7	123.6	25.4	−22.5	58.1	63.9	252.7	4.8
PLA/30SCG	144.2	153.9	100.0	24.8	−17.7	56.7	64.8	149.5	155.4	123.9	20.5	−16.6	57.5	56.6	230.7	8.4

**Table 2:** ANOVA test for impact results of PLA and its composites.

Source of Variation	SS	Df	MS	F	*p*-Value	F_critical_
AG	450.254	3	150.0847	**8.396984**	**0.000543**	**3.08787**
WG	428.9673	24	17.87364			
Total	879.2214	27				

Note: AG—among groups; WG—within groups; Df—degrees of freedom; SS—sum of squares; MS—mean square; F—F-test for one-way ANOVA; Number of observations = 7; Number of samples = 4.

## Data Availability

Not applicable.
